# Supplementary material for: Characteristics of the First Domestic Duck-Origin H12N8 Avian Influenza Virus in China
Source: Int J Mol Sci. 2025 Mar 18;26(6):2740. doi: 10.3390/ijms26062740 (PMC11943133; doi:10.3390/ijms26062740)
Supplement: Supplementary file 1 [file ijms-26-02740-s001.zip › Supplementary tables/Zhao table S1.pdf]

Table S1 Global prevalence of H12 AIVs.

Host	Number	Subtype*
Ruddy turnstone	187	H12Ny, H12N1, H12N2, H12N3, H12N4, H12N5, H12N6, H12N7, H12N8, H12N9
Mallard	135	H12Ny, H12N1, H12N2, H12N4, H12N5, H12N7, 12N8, H12N9
Duck	43	H12Ny, H12N1, H12N2, H12N5, H12N6, H12N7, H12N8
Northern Pintail	18	H12Ny, H12N2, H12N5, H12N8
Blue-winged teal	12	H12Ny, H12N2, H12N3, H12N4, H12N5, H12N7
Red knot	11	H12Ny, H12N3, H12N4, H12N5, H12N9
Common teal	9	H12N2, H12N5, H12N8
Environment	10	H12Ny, H12N2, H12N5, H12N7
Pintail	9	H12Ny, H12N5
Northern shoveler	7	H12Ny, H12N2, H12N5
Teal	6	H12N2, H12N5, H12Ny
American green-winged teal	4	H12N2, H12N4, H12N5
Laughing gull	4	H12N4, H12N5, H12N6
American wigeon	4	H12N5
Australian shelduck	3	H12N5
Gadwall	3	H12Ny, H12N5
Green-winged teal	6	H12N3, H12N5
Lesser whistling duck	3	H12N1
Shoveler	3	H12N5
Common goldeneye	4	H12N5
Ruddy shelduck	2	H12N3
Surf scoter	2	H12N5
Wild duck	2	H12N5, H12N8
Yellow-billed pintail	2	H12N5
Spotbill duck	2	H12N6
American black duck	1	H12N5
Arenaria interpres	1	H12N3
Bar-headed goose	1	H12N1
Bewick swan	1	H12N5
Bufflehead	1	H12N5
Common eider	1	H12N5
Common murre	1	H12N1
Cygnus Olor	1	H12N6
Dunlin	1	H12N5

Host	Number	Subtype*
Eurasian wigeon	1	H12N5
Garganey	1	H12Ny
Guinea fowl	1	H12N2
Lesser flamingo	1	H12Ny
Mareca falcata	1	H12Ny
Muscovy duck	1	H12N2
Ostrich	1	H12N8
Ring-necked duck	1	H12N7
Sanderling	1	H12N4
Semipalmated sandpiper	1	H12N4
Slaty-backed gull	1	H12N5
Swan	1	H12N2
Thick-billed murre	1	H12N5
Watercock	1	H12N1
White-winged scoter	1	H12N5
Whooper swan	1	H12N3
Yellow-billed teal	1	H12N5
Unknown#	14	H12N5, H12Ny

The information of H12 AIVs were downloaded from GISAID and GenBank database.

*The H12Ny virus in the subtype column indicates that the NA of the virus was not determined.

#The unknown hosts of the H12 viruses include aquatic bird, shorebird, wild bird, wild waterbird, wild migratory bird, and feces.
