# Supplementary material for: Characteristics of the First Domestic Duck-Origin H12N8 Avian Influenza Virus in China
Source: Int J Mol Sci. 2025 Mar 18;26(6):2740. doi: 10.3390/ijms26062740 (PMC11943133; doi:10.3390/ijms26062740)
Supplement: Supplementary file 1 [file ijms-26-02740-s001.zip › Supplementary tables/Zhao table S2.pdf]

Table S2. The potential glycosylation site of HA and NA protein of the DK/FJ/D62/2020 virus.

Protein	Potential glycosylation site	Amino acid
HA	27	NNS
	28	NST
	140	NVT
	151	NDT
	222	NRS
	302	NTS
	309	NTS
	496	NGT
	523	NST
NA	46	NGT
	54	NET
	84	NNT
	144	NGT
	293	NWT
	398	NWS
