# Supplementary material for: Characteristics of the First Domestic Duck-Origin H12N8 Avian Influenza Virus in China
Source: Int J Mol Sci. 2025 Mar 18;26(6):2740. doi: 10.3390/ijms26062740 (PMC11943133; doi:10.3390/ijms26062740)
Supplement: Supplementary file 1 [file ijms-26-02740-s001.zip › Supplementary tables/Zhao table S3.pdf]

Table S3. The highest percentage of nucleotide identity of the DK/FJ/D62/2020 virus using BLAST method in GISAID and NCBI database.

Gene	The highest similarity strain	Similarity of nucleotide	Accession number
HA	A/duck/Vietnam/G18/2009 (H12N5)	94.93%	EPI315147
NA	A/Environment/Fujian/85144/2014 (H1N8)	97.38%	EPI1315730
PB2	A/environment/Fujian/EV01/2020 (H11N3)	99.25%	EPI2162132
PB1	A/Environment/Fujian/44488/2019 (H3N2)	98.02%	EPI2211285
PA	A/duck/Japan/AQ-HE103/2015 (H1N2)	97.91%	EPI1015036
NP	A/Fujian/33845/2017 (H7N9)	98.67%	EPI1252144
M	A/environment/Fujian/EV01/2020 (H11N3)	99.08%	EPI2162126
NS	A/duck/Zhejiang/S4489/2014 (H7N9)	99.20%	EPI1090793
